# Changes in Blood Potassium after Reperfusion during Living-Donor Liver Transplantation: An Exploratory Study

**DOI:** 10.3390/diagnostics11122248

**Published:** 2021-11-30

**Authors:** Jeayoun Kim, Ji-Hye Kwon, Gaab Soo Kim

**Affiliations:** Department of Anesthesiology and Pain Medicine, Samsung Medical Center, Sungkyunkwan University School of Medicine, Seoul 06351, Korea; jeayoun.kim@samsung.com (J.K.); jh0828.kwon@samsung.com (J.-H.K.)

**Keywords:** living donor liver transplantation, hyperkalemia, reperfusion, potassium

## Abstract

The incidence of hyperkalemia (>5.5 mEq/L) or high blood potassium (5–5.5 mEq/L) during living-donor liver transplantation (LDLT) is reported to be more than 10%. It occurs more frequently in the early post-reperfusion period and is a major cause of post-reperfusion arrhythmia and cardiac arrest. Unlike deceased-donor liver transplantation, the pattern of blood potassium changes immediately after reperfusion has not been described in LDLT. From January 2021 to March 2021, fifteen consecutive patients were enrolled. Baseline blood potassium was measured from blood samples obtained 10-min (T_-10_) and immediately before (T_0_) reperfusion. During the first 5 min after reperfusion, blood potassium measurements were conducted every one minute (T_1_–T_5_). The blood potassium levels at T_-10_ and T_0_ were 3.8 ± 0.4 and 3.9 ± 0.4 mEq/L, respectively. After reperfusion, mean increases (95% CI) in blood potassium from T_-10_ and T_0_ were 0.5 (0.4–0.6) and 0.4 (0.3–0.5) mEq/L, respectively. Blood potassium peaked at T_1_, returned to baseline at T_3_, and fell below the baseline at T_5_. Peak blood potassium after reperfusion showed strong correlations with blood potassium measured at T_-10_ (*p* < 0.001) and T_0_ (*p* < 0.0001). These findings can support the establishment of future research plans and perioperative management of blood potassium in LDLT.

## 1. Introduction

Liver transplantation (LT) is a well-established therapeutic option for patients with end-stage liver disease or hepatocellular carcinoma. The complex surgical procedure and patients’ underlying conditions are frequently associated with significant morbidity and mortality. Derangement in blood potassium is an important issue during LT [1,2]. Elevated blood potassium is a major cause of post-reperfusion arrhythmia and cardiac arrest [2,3,4]. The occurrence of hyperkalemia (>5.5 mEq/L) or high blood potassium (5–5.5 mEq/L) is more frequent in the early post-reperfusion period than at other times [5,6]. 

Studies evaluating blood potassium after reperfusion have mainly been conducted in deceased-donor liver transplantation (DDLT), not living-donor liver transplantation (LDLT) [6,7]. The lack of evidence and concern about post-reperfusion hyperkalemia could lead clinicians to try to decrease blood potassium with preemptive medication before reperfusion. Considering the substantial differences between DDLT and LDLT, studying the changes in blood potassium during LDLT is warranted. Therefore, the present study verified changes in blood potassium after reperfusion during LDLT.

## 2. Materials and Methods

This study is a prospective observational study conducted at Samsung Medical Center, a quaternary level university teaching hospital. This study was approved by the Samsung Medical Center Institutional Review Board (IRB no. SMC 2020-12-087) and registered with the Clinical Trial Registry of Korea in December 2020 (KCT0005754, https://cris.nih.go.kr; principal investigator: Gaab Soo Kim; date of registration: 8 January 2021) before recruitment of the first participant. 

Consecutive adult patients (older than 18 years) scheduled for elective LDLT from 11 January 2021, to 30 March 2021, were screened. After written informed consent was obtained, patients were assessed for eligibility and enrolled in this prospective study. Exclusion criteria were age older than 75 and multi-organ transplantation.

### 2.1. Anesthetic and Surgical Management

We performed standardized anesthesia under our institutional LT protocol. After patients arrived in the operating room, we applied standard monitors, including 5-lead electrocardiography, noninvasive blood pressure, and pulse oximetry. After endotracheal intubation, catheterization of the radial artery, femoral artery, and femoral vein was conducted. A 9Fr catheter (Teleflex, Wayne, PA, USA) and pulmonary artery catheter (Swan Ganz SvO_2_, Edwards, Irvine, CA, USA) were inserted through the internal jugular vein to manage fluid and vasoactive-inotropic agents. 

We mainly used Plasma Solution A (CJ Healthcare, Seoul, Korea) as the crystalloid, which has the same composition as Plasma-Lyte 148 solution (Baxter Healthcare, Toognabbie, NSW, Australia). It is a balanced salt solution with a composition similar to serum with potassium (5 mEq/L). Five percent albumin (Green Cross Corp., Seoul, Korea), Hextend^®^ (BioTime, Berkeley, CA, USA), or Volulyte^®^ (Fresenius Kabi, Bad Homburg, Germany) were used for colloid intervention. Hextend^®^ and Volulyte^®^ include 3 mEq/L and 4 mEq/L of potassium, respectively. If the blood potassium exceeded 4.5 mEq/L during the preanhepatic or anhepatic phase, the attending clinician administrated a bolus of 5 or 10 international units of regular insulin with 200 mL of 5% dextrose solution. Our institutional protocol for blood potassium management is similar to those reported in previous research [1,8]. Electrolytes and arterial blood gases were monitored throughout surgery and corrected as needed. At the end of surgery, recipients were transported to an intensive care unit for postoperative care.

The transfusion policy of our transplant team is characterized by restrictive use of blood products. Each blood component is separately transfused based on its respective indication. Intraoperatively, the target blood hemoglobin for allogeneic RBCs was 8.0 g/dL. Blood salvage is routine procedure during surgery to reduce the exposure to allogeneic RBCs. Salvaged blood was primarily transfused when hemoglobin was lower than 9 g/dL. We transfused fresh frozen plasma to maintain the prothrombin time, expressed as an international normalized ratio, below 3 and platelets to keep the platelet count above 30,000/µL. Additionally, we transfused cryoprecipitates when fibrinogen fell below 80 mg/dL.

All procured grafts were preserved with histidine-tryptophan-ketoglutarate (HTK) solution (Custodiol, Alsbach, Germany). No additional rinsing was done through the portal vein or hepatic artery. Caval anastomosis was performed using the piggyback technique with or without a temporary portocaval shunt. Before starting and completing the portal vein anastomosis, the surgeon drained about 100 mL of blood through the recipient’s portal vein to remove congested blood and potential thrombi.

### 2.2. Study Protocol

The blood samples were obtained from a pre-placed radial artery catheter using heparinized syringes and analyzed by a blood gas analyzer (RAPIDLab 1200 Blood Gas Analyzer; Siemens Healthcare, Erlangen, Germany). A board-certified anesthesiologist acquired, transported, and analyzed all blood samples to minimize any bias, including in vitro hemolysis and inter-operator variability.

Blood samples were acquired 10-min (T_-10_) and immediately (T_0_) before reperfusion to measure the baseline blood potassium. During the first 5 min after reperfusion, blood samples were acquired every one minute (T_1_–T_5_). Moreover, electrocardiography was analyzed during the reperfusion period. We did not administer medications that might affect the blood potassium (sodium bicarbonate, insulin, dextrose solution, or bolus catecholamines, etc.) just before reperfusion [9].

The primary outcome was the change in blood potassium during the first 5 min after reperfusion. We also investigated correlations between the peak blood potassium after reperfusion and perioperative variables: the model for end-stage liver disease (MELD) score, warm ischemic time (WIT), cold ischemic time (CIT), graft to recipient weight ratio, blood potassium levels measured preoperatively and before reperfusion, and vasoactive-inotropic score at reperfusion [10]. We also investigated the correlation between hemodynamic parameters and laboratory values one hour after portal clamping and between T_-10_ and peak blood potassium [5].

### 2.3. Statistical Analysis

The categorical variables are presented as the numbers and percentages (%). We conducted the Shapiro–Wilk test to assess normality. Continuous variables are presented as the mean ± standard deviation (SD) when the data were normally distributed and as the median (interquartile rage (IQR)) when the distribution was skewed. We used paired t-testing with Bonferroni correction for multiple comparisons to evaluate blood potassium changes from the baseline to the pre-specified time points. The strength of the associations between peak blood potassium and perioperative variables are presented using Pearson correlation coefficients. Statistical analyses were performed using SAS version 9.4 (SAS Institute, Cary, NC, USA) and SPSS version 27 (IBM, Chicago, IL, USA), and *p* < 0.05 was considered statistically significant.

## 3. Results

Among the 23 patients who were screened for this trial, eight patients declined to participate. Therefore, 15 patients were enrolled and completed this study. Their preoperative characteristics are presented in Table 1. Their preoperative blood potassium was 4.0 ± 0.4 mEq/L. The MELD scores ranged between 8 and 38, with a median (IQR) of 9.0 (7.0–13.5), and hemoglobin was 11.0 ± 2.6 g/dL. Their creatinine was 0.67 (0.60–0.97) mg/dL, and their estimated glomerular filtration rate was 93.1 ± 26.7 mL/min/1.73 m^2^. Two of the patients had chronic kidney disease with high preoperative creatinine (patient 1: 2.37 mg/dL, patient 2: 1.14 mg/dL). Intraoperative variables are summarized in Table 2. The WIT and CIT were 44.1 ± 14.1 and 103.8 ± 36.5 min, respectively. The anhepatic phase was 156.5 ± 36.1 min. Blood potassium was 3.8 ± 0.4 mEq/L at T_-10_ and 3.9 ± 0.4 mEq/L at T_0_. Before reperfusion, 5 patients (33.3%) received 2.0 (1.0–2.0) units of allogeneic RBCs. Vasoactive drugs were used as a continuous infusion and not a bolus administration. At the time of reperfusion, 8 patients (53.3%) were receiving norepinephrine of 0.1 (0.05–0.15) mcg/kg/min, and 4 patients (26.7%) were receiving dopamine of 5.00 (4.50–6.25) mcg/kg/min. Postreperfusion syndrome occurred in 7 patients (46.7%).

### 3.1. Primary Outcomes

**Figure 1:**
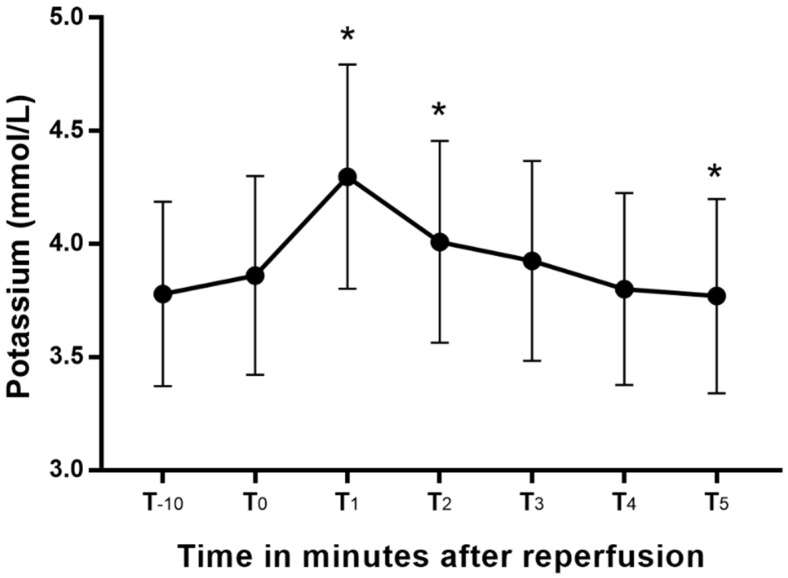
Blood potassium changes after reperfusion presented as a box-whisker plot. *: *p* < 0.05 versus T_0_.

Blood potassium changed significantly from the values at T_0_ (Figure 1), peaking at T_1_. Blood potassium at T_1_ and T_2_ were significantly greater than the T_0_, and that at T_5_ was significantly lower than the blood potassium at T_0_. The mean increases (95% CI) in blood potassium from T_-10_ and T_0_ to peak blood potassium were 0.5 (0.4–0.6) mEq/L and 0.4 (0.3–0.5) mEq/L, respectively. The two patients with chronic kidney disease had blood potassium levels higher than 4.5 mEq/L during the anhepatic period, and they were treated with 10 units of regular insulin and 200 mL of 5% dextrose solution. After that treatment, their blood potassium decreased from 6.6 to 4.7 mEq/L in patient 1 and 4.7 to 4 mEq/L in patient 2. Patient 1 developed high blood potassium (5.33 mEq/L) after reperfusion. However, no additional treatment was conducted, and the electrocardiography implied an absence of hyperkalemia. 

### 3.2. Other Outcomes

**Figure 2:**
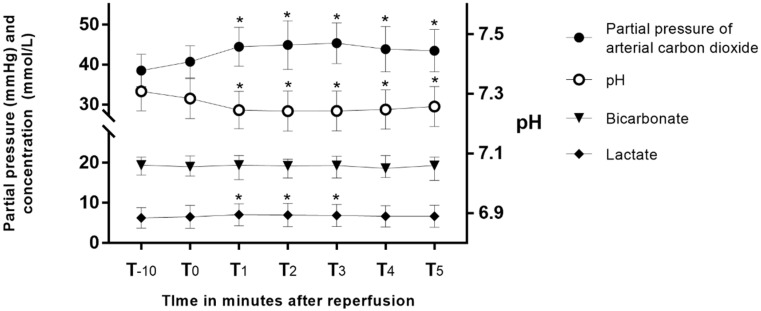
Changes in partial pressure of arterial carbon dioxide, pH, bicarbonate, and lactate after reperfusion. *: *p* < 0.05 versus T_0_.

Peak blood potassium after reperfusion showed strong correlations with preoperative blood potassium (Pearson’s r = 0.7, *p* < 0.004) and blood potassium at T_-10_ (Pearson’s r = 0.97, *p* < 0.001) and T_0_ (Pearson’s r = 0.95, *p* < 0.0001). Other perioperative variables did not show a significant correlation with peak blood potassium (Table 3). Hemodynamic parameters and laboratory values measured one hour after the anhepatic phase and at T_-10_ are presented in Supplementary Table S1. They did not show a significant relationship with peak blood potassium. Changes in the partial pressure of arterial carbon dioxide (PaCO_2_), pH, bicarbonate, and lactate after reperfusion are presented in Figure 2. After reperfusion, PaCO_2_ increased, and pH decreased significantly, and those values did not return to baseline after 5 min. Lactate increased until T_3_ and then returned to baseline at T_4_. After reperfusion, mean arterial blood pressure started to decrease at T_2_ and returned to its baseline at T_4_ (Supplementary Figure S1).

## 4. Discussion

Our main finding in this study is that blood potassium peaked one minute after reperfusion and then fell below its baseline value within 5 min. The mean changes (95% CI) in blood potassium from T_-10_ and T_0_ to peak blood potassium were 0.5 (0.4–0.6) mEq/L and 0.4 (0.3–0.5) mEq/L, respectively. Peak blood potassium had a strong correlation with pre-reperfusion blood potassium levels. To our knowledge, this is the first study to repetitively check blood potassium at short intervals immediately after reperfusion in LDLT.

Hyperkalemia occurs frequently in the early postreperfusion period [6]. When a graft is reperfused, the release of congested blood can cause marked acidemia that mobilizes potassium from a large intracellular pool [1,2]. Our data also show a significant decrease in pH after reperfusion. Moreover, the liver can release large amounts of intracellular potassium during stressful conditions [11], and preservation solutions with a high potassium concentration can also lead to acute hyperkalemia [1,12]. Two comprehensive studies investigated the incidence, etiology, and outcome of intraoperative cardiac arrest (ICA) during adult LT [3,4]. They reported that ICA occurred most frequently within 5 min after reperfusion, and patients with ICA showed higher mortality than other LT patients. Interestingly, hyperkalemia was the most common cause of ICA in that predominantly LDLT study population [4]. Therefore, vigilant monitoring of blood potassium during the reperfusion period is as important in LDLT as it is in DDLT.

We showed that the trend in blood potassium after reperfusion in LDLT was similar to that in DDLT. In one DDLT study, blood potassium was measured at 30 and 90 s and 5 min after reperfusion. The blood potassium in that study peaked at 30 s and fell below the baseline at 5 min [13]. Weinberg et al. more accurately measured blood potassium in DDLT using 13 time points during the first 5 min after reperfusion. They discovered that blood potassium peaked 80 s after reperfusion, plateaued for a further minute, and then returned toward baseline values at 5 min [7]. Those results suggest that measuring blood potassium one or two times after reperfusion is not enough to find the exact time when it peaks. In LDLT, the incidence and risk factors for high potassium have been investigated. However, those researchers monitored blood potassium only five times during the entire operation and did not clarify the exact time points [14]. We prospectively measured blood potassium in LDLT at one-minute intervals immediately after reperfusion. Our results show that blood potassium peaked in 1 min, plateaued until 2 min, and then fell below the baseline value at 5 min, which has implications for monitoring hyperkalemia during the reperfusion phase. Blood potassium checked 2 min after reperfusion might not accurately reflect the peak blood potassium and could underestimate the risk of unwanted cardiac events and arrhythmia.

Our results differed from results in DDLT in the incidence and magnitude of blood potassium changes. In a retrospective study of 1124 adult recipients of mostly (96.2%) DDLT, the incidence of hyperkalemia after reperfusion was 19.1% [6]. In LDLT, 10.4% of 487 patients had at least one episode of high blood potassium during surgery [14]. The incidence of high blood potassium (1/15, 6.7%) in this study was lower than in DDLT, despite the low threshold for diagnosing elevated blood potassium, and was closer to previous LDLT studies. Further, the increase in blood potassium after reperfusion was less than one-third of the changes seen in DDLT: 0.5 (0.4–0.6 range 0.4–0.7) vs. 1.5 (1.3–1.7 range 1.1–3.0) [13]. Differences between the grafts are thought to explain the lower incidence and magnitude of hyperkalemia in LDLT. For example, the median CIT in this study was 98.0 (79.0–115.0) minutes, more than 4 times shorter than in previous DDLT studies [3,6]. The short CIT of LDLT could minimize the preservation injury and reduce ischemia-reperfusion injury [15], which contributes to the lower incidence and reduces the magnitude of hyperkalemia. Moreover, a deceased donor might have been exposed to hypoperfusion in a hypovolemic state or the use of a high dose vasopressor before graft donation [3,4,16]. Accordingly, intraoperative complications, especially hyperkalemia, are more likely occur in DDLT than LDLT [6]. In addition to the difference between living and deceased donors, preservation solutions can affect changes in blood potassium. Previous studies investigating blood potassium in LT either did not specify the preservation solution [6,13,14] or used University of Wisconsin solution [5,7,16]. We used HTK solution in this study, and its potassium content (9 mEq/L) is lower than that of University of Wisconsin solution (125 mEq/L). The type of preservative solution might result in different patterns of hyperkalemia [17,18]. We suggest that measuring blood potassium at T_-10_ gives clinicians time to intervene in the occurrence of reperfusion hyperkalemia. Moreover, we think it is better to maintain the threshold of 4.5 mEq/L for pre-emptive treatment in LDLT based on the results of this study.

In a study evaluating risk factors for hyperkalemia in LT, post-reperfusion hyperkalemia correlated more with insufficient cardiac output and decreased liver lactate uptake during the anhepatic phase than with the duration of cold ischemia or preservation injury of the graft [5]. Unfortunately, we could not find any significant relationship between blood potassium and cardiac output, base excess, or lactate during the anhepatic phase. Our results reveal that pre-reperfusion blood potassium had a strong association with peak blood potassium after reperfusion, but other intraoperative variables failed to show a significant association with peak blood potassium; this result is in line with previous studies [5,6,14]. Two of our patients were treated with regular insulin and a dextrose solution during the anhepatic period according to our institutional protocol. Both patients had chronic kidney disease with preoperative creatinine elevation. Despite treatment before reperfusion, one of them showed high potassium during reperfusion. Although changes in blood potassium in LDLT might be less than those in DDLT, patients with kidney dysfunction and other high-risk recipients might require blood potassium management before reperfusion to prevent unwanted cardiac events. 

Our study has several limitations. This was a single-center study, and our anesthetic and surgical management during LT might differ from those in other institutions, limiting the external validity of our findings. For example, we did not administer preemptive drugs immediately before reperfusion; using those drugs could produce a pattern different from our results. Additionally, surgeons in our center drained about 100 mL of blood two times through the recipient’s portal vein before reperfusion. We assume that process might help to remove inflammatory metabolites and endotoxins of the splanchnic bed that accumulated during the anhepatic phase [19]. Reperfusion techniques unique to each hospital might influence changes in blood potassium after reperfusion. Furthermore, our sample size (*n* = 15) was too small to discover significant risk factors other than pre-reperfusion blood potassium, and we could not clarify the effect that elevated blood potassium had on clinical outcomes. Considering those limitations, further studies are needed. Nonetheless, our findings might be helpful in making hypotheses, and they provide valuable data for determining the sample size for future studies. 

## 5. Conclusions

In LDLT, blood potassium peaks 1 min after reperfusion and falls below the baseline blood potassium level within 5 min. The mean increase in blood potassium was 0.4 mEq/L above baseline (T_0_). These findings might help in the perioperative management of blood potassium in LDLT.

## Figures and Tables

**Table 1:** Preoperative characteristics.

Variable	Result	Normal Range
Male, *n* (%)	11 (73.3)	
Age, years, mean ± SD	56.1 ± 10.0	
Body Mass Index, kg/m^2^, mean ± SD	25.1 ± 4.7	
Chronic kidney disease, *n* (%)	2 (13.3)	
Hypertension, *n* (%)	4 (26.7)	
Diabetes mellitus, *n* (%)	5 (33.3)	
ABO incompatible liver transplantation, *n* (%)	4 (26.7)	
MELD score, median (IQR)	9.0 (7.0–13.5)	
**Indications for liver transplantation**		
Liver cirrhosis, *n* (%)	5 (33.3)	
Hepatocellular carcinoma, *n* (%)	9 (60.0)	
Acute liver failure, *n* (%)	1 (6.7)	
**Preoperative laboratory results**		
Hemoglobin, g/dL, mean ± SD	11.0 ± 2.6	male 13.6–17.4 female 11.2–14.8
Hematocrit, %, mean ± SD	33.0 ± 7.5	male 40.4–51.3 female 31.8–43.8
White blood cell count, ×10^3^/μL, median (IQR)	3.6 (2.7–6.2)	male 3.80–10.58 female 3.15–8.63
Platelets, ×10^3^/μL, mean ± SD	101.9 ± 58.3	male 141–316 female 138–347
International Normalized Ratio, mean ± SD	1.3 ± 0.3	0.9–1.1
Sodium, mmol/L, median (IQR)	139.0 (138.0–141.0)	136–145
Potassium, mmol/L, mean ± SD	4.0 ± 0.4	3.5–5.1
Chloride, mmol/L, median (IQR)	105.0 (103.0–107.0)	98−107
Blood urea nitrogen, mg/dL, median (IQR)	13.3 (11.7–20.6)	6–23
Creatinine, mg/dL, median (IQR)	0.67 (0.60–0.97)	0.70–1.20
Estimated GFR, mL/min/1.73 m^2^, mean ± SD	93.1 ± 26.7	60–150
Calcium, mg/dL, mean ± SD	8.4 ± 0.4	8.6–10.2
Magnesium, mg/dL, mean ± SD	1.9 ± 0.2	1.6–2.6
Phosphate, mg/dL, mean ± SD	3.9 ± 0.6	3.0–4.5
Bilirubin, mg/dL, median (IQR)	1.0 (0.7–1.9)	0–1.2
Alanine transaminase, U/L, mean ± SD	29.0 ± 17.4	0–40
Aspartate transaminase, U/L, median (IQR)	33.0 (28.5–39.5)	0–41
Gamma-glutamyl transferase, U/L, mean ± SD	75.9 ± 45.9	10–71
Alkaline phosphatase, U/L, mean ± SD	102.1 ± 47.4	40–129
Total protein, g/dL, mean ± SD	6.2 ± 0.7	6.4–8.3
Albumin, g/dL, mean ± SD	3.2 ± 0.6	3.5–5.2

Continuous data were presented as mean ± standard deviation when normally distributed or median [interquartile rage] when skewed distributed. Categorical data were presented as number (proportion). Abbreviations: MELD score, model for end-stage liver disease score; GFR, glomerular filtration rate.

**Table 2:** Intraoperative variables. Preoperative characteristics.

Variable	Result
GRWR, mean ± SD	1.12 ± 0.3
Warm ischemia time, min, mean ± SD	44.1 ± 14.1
Cold ischemia time, min, mean ± SD	103.8 ± 36.5
Postreperfusion syndrome, *n* (%)	7 (46.7)
Transient portocaval shunt, *n* (%)	1 (6.7)
**Surgery stage**	
Total operative time, min, mean ± SD	418.3 ± 71.6
Dissection, min, median (IQR)	62.0 (53.0–71.5)
Anhepatic, min, mean ± SD	156.5 ± 36.1
Neohepatic, min, mean ± SD	191.7 ± 47.0
**Prereperfusion blood potassium**	
10 min before reperfusion, mmol/L, mean ± SD	3.8 ± 0.4
Immediately before reperfusion, mmol/L, mean ± SD	3.9 ± 0.4
**Fluid before reperfusion**	
Plasma solution A, *n* (%)	15 (100.0)
Volume, mL/kg, mean ± SD	34.8 ± 14.8
5% Albumin, *n* (%)	15 (100.0)
Volume, mL/kg, mean ± SD	7.1 ± 2.3
Hextend, *n* (%)	9 (60.0)
Volume, mL/kg, median (IQR)	7.2 (6.1–9.7)
Volulyte, *n* (%)	7 (46.7)
Volume, mL/kg, median (IQR)	9.4 (7.7–12.7)
**Transfusion before reperfusion**	
Allogeneic RBC, *n* (%)	5 (33.3)
Number of unit, median (IQR)	2.0 (1.0–2.0)
Platelet, *n* (%)	0 (0.0)
Fresh frozen plasma, *n* (%)	1 (6.7)
Cryoprecipitate, *n* (%)	0 (0.0)
Infused salvaged blood, *n*(%)	5 (33.3)
Total volume, mL, median (IQR)	291.0 (278–300)
**Vasoactive drugs at reperfusion**	
VIS, median (IQR)	5.0 (0.0–10.0)
Norepinephrine, *n* (%)	8 (53.3)
Dose, mcg/kg/min, median (IQR)	0.10 (0.05–0.15)
Dopamine, *n* (%)	4 (26.7)
Dose, mcg/kg/min, median (IQR)	5.00 (4.50–6.25)
Vasopressin, *n* (%)	1 (6.7)
Dose, unit/hr, median (IQR)	1.2 (1.2–1.2)

Continuous data were presented as mean ± standard deviation when normally distributed or median (interquartile range) when skewed distributed. Categorical data were presented as number (proportion). Abbreviations: MELD score, model for end-stage liver disease score; GRWR, graft to recipient weight ratio; VIS, Vasoactive-Inotropic Score.

**Table 3:** Correlation analysis of perioperative factors associated with peak blood potassium after reperfusion.

	Correlation Coefficient	Raw *p*-Value
MELD score	0.20	0.47
Preoperative potassium values, mmol/L	**0.70**	**0.004**
GRWR	0.15	0.59
Warm ischemia time, min	−0.02	0.94
Cold ischemia time, min	0.07	0.79
Total operative time, min	0.15	0.59
Anhepatic time, min	−0.02	0.95
**Prereperfusion blood potassium**		
10 min before reperfusion, mmol/L	**0.97**	**<0.0001**
Immediately before reperfusion, mmol/L	**0.95**	**<0.0001**
**Fluid and blood products before reperfusion**		
Allogeneic RBC, unit	0.34	0.22
Salvaged blood, mL	0.27	0.33
Plasma solution A, mL/kg	−0.01	0.97
Albumin (5%), mL/kg	0.09	0.75
Volulyte, mL/kg	0.27	0.77
Hextend, mL/kg	−0.29	0.29
Vasoactive-Inotropic Score	−0.17	0.55

Abbreviations: MELD score, model for end-stage liver disease score; GRWR, graft to recipient weight ratio. The bold means significant correlation with peak blood potassium after reperfusion.

## Data Availability

Not applicable.

## Supplementary Materials

The following are available online at https://www.mdpi.com/article/10.3390/diagnostics11122248/s1. Figure S1: Changes in mean arterial blood pressure after reperfusion. Values are the median (interquartile range). *: *p* < 0.05 versus baseline. Table S1: Correlation analysis of factors associated with peak blood potassium after reperfusion. Continuous data were presented as mean ± standard deviation when normally distributed or median [interquartile rage] when skewed distributed. Categorical data were presented as number (proportion).
