# Supplementary material for: Changes in Blood Potassium after Reperfusion during Living-Donor Liver Transplantation: An Exploratory Study
Source: Diagnostics (Basel). 2021 Nov 30;11(12):2248. doi: 10.3390/diagnostics11122248 (PMC8700509; doi:10.3390/diagnostics11122248)
Supplement: Supplementary file 1 [file diagnostics-11-02248-s001.zip › diagnostics-1476297-supplementary.pdf]

Supplementary Table S1. Correlation analysis of factors associated with peak blood potassium after reperfusion. Continuous data were presented as mean ± standard deviation when normally distributed or median [interquartile rage] when skewed distributed.

	1-hour after anhepatic phase	Correlation coefficient	Raw p-value	10-minute before reperfusion	Correlation coefficient	Raw p-value
**Hemodynamics parameters**						
Heart rate	89.2 ± 17.4	-0.14	0.61	93.5 ± 4.2	-0.24	0.4
Mean ABP	93.9 ± 11.1	0.3	0.29	77.1 ± 11.3	0.18	0.53
CVP	4.1 ± 2.9	-0.2	0.47	4.2 ± 2.6	0.08	0.77
Mean PAP	11.0 [10.5-13.0]	-0.3	0.32	11.0 [10.0-14.5]	-0.3	0.29
PCWP	6.0 [5.0-8.0]	-0.2	0.45	6.0 [6.0-8.8]	0.07	0.82
CI	3.7 ± 1.5	0.24	0.39	4.0 ± 1.7	0.15	0.59
SVR	1100.6 ± 446.2	0.22	0.43	968.4 ± 472.5	0.19	0.49
RVEF	37.6 ± 7.7	0.28	0.32	38.2 ± 7.9	-0.29	0.29
**Laboratory values**						
pH	7.35 ± 0.05	0.01	0.98	7.31 ± 0.07	0.2	0.48
PaCO2	39.8 [36.8-41.0]	-0.12	0.68	38.5 ± 1.0	0.37	0.17
PaO2	280.4 ± 32.1	0.43	0.11	274.7 ± 30.9	0.05	0.86
Base excess	-4.5 ± 3.3	0.11	0.89	-6.7 ± 3.5	-0.17	0.57
Lactate	4.49 ± 1.87	-0.31	0.27	6.27 ± 2.55	-0.29	0.29

Abbreviations: ABP, arterial blood pressure; CVP, central venous pressure; PAP, pulmonary artery pressure; PCWP, pulmonary capillary wedge pressure; CI, cardiac index; SVR, systemic vascular resistance; PaCO2, partial pressure of arterial carbon dioxide; PaO2, partial pressure of arterial oxygen.

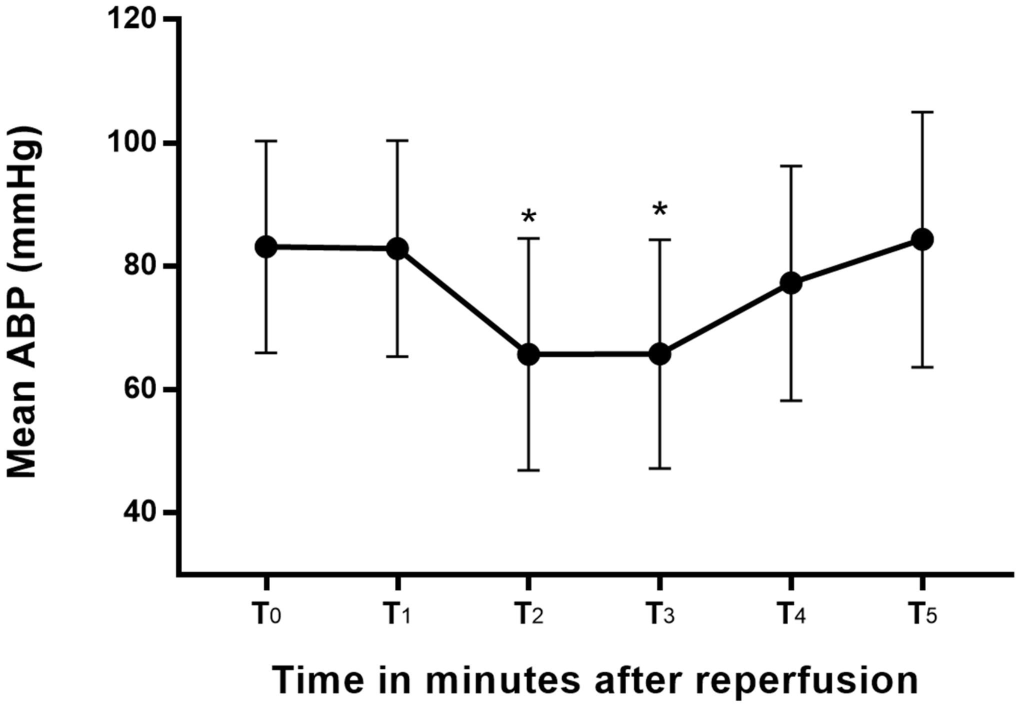

Supplementary Figure S1. Changes in mean arterial blood pressure after reperfusion.

Values are the median (interquartile range). *: $p < 0.05$ versus baseline.
